# A Great Want

**Published:** 1861-10

**Authors:** 


### A GREAT WANT.

Parents who do not exercise a careful supervision over the reading matter of their children, omit a duty of vital importance, and may reasonably anticipate subsequent disappointment, mortification, and sorrow, in the failure of those children to meet the expectations which had been formed of them. Aaron Burr revelled in the reading of infidel books in early youth; and yet, with talents to have made him a second Washington, he went down to his grave with the reputation of a corrupter of his kind, a traitor, and a murderer.

The son of the immortal John Howard, the friend of man, with all the advantages of a superior education and high social position, left to himself, to read what he listed—his mother being dead, and his father in foreign lands—fell into debauchery, and died a drunken madman, in the lunatic asylum of Leicester, before he was thirty-five.

It is recorded of the Emperor Paul, the Nero of modern times, one of the most execrable of men, if received histories are true, that he took the utmost delight in reading horrible tales of every description, in contemplating pictures of rapine, murder, and blood, only to practice them all when, a little later, he was placed on the throne of all the Russias.

Children can read and hear moral poison; and moral death is as certain to follow, as will physical death, if the poison of the apothecary is swallowed. A grain of strychnine is not the less fatal from being enveloped in sugar, or mingled with a hundred times its bulk in a teaspoonful of molasses.

A "splendid lecture" from a "splendid man," is rather the more pernicious, from having a single idea—atheistic, infidel, or impure; and a newspaper, a monthly, or a quarterly, may be, in the main, conducted with singular ability; but if a *roue*, or pantheist, or deist, have it under control, its very ability only adds to the malignancy of occasional propositions, in more or less direct consonance with the individual sentiments of the editor. We undertake to say, that this occasional jactatory exhibition of sentiments, radically subversive of moral purity and true religion, is a part and parcel of the design of too many of the monthly magazines and city newspapers, to some of which there is no responsible name—no distinct editor; while many of the various writers, taking advantage of their impersonality, shoot in the dark, and stab from behind, with the spirit of an assassin and the malignity of a demon.

An exchange says of a certain lecturer, and a most popular contributor to a magazine, which is loudly lauded every month by too many religious newspapers, for getting it for nothing as an exchange, and for the real good things that are in its pages: "In his lecture, he opposed the idea that a large part of mankind are to be eternally damned, and used the expression, in describing the gallant act of a fireman:

"'He knows full well, from what he was taught on his mother's knees, that, if he perishes, he will go to a place so hot that all the fire-engines in the world cannot give him a drop of water to cool his parched tongue.'"

On the above, another paper remarks: "We can give no language to express our sense of the outrage committed on the religious feelings of the audience, crashing through all the finest sensibilities of his hearers, as unconscious, apparently, of what he was doing, as a wild boar tearing through a garden of flowers."

Any man who could have the effrontery thus ruthlessly to insult, by an impious jest, the religious sentiments of a majority of his audience, and of his country, is unfit to write for the families of that majority; and yet, for a long time, this lecturer has been the most attractive feature in one of the most popular monthlies in the country, and which has other contributors to shoot out their infidelities, as occasion offers.

"A great want" is it, then, to have a monthly publication visit our families, which will displace such a "literature," as it is inaptly termed; a monthly contributed to by men of better hearts, of sounder heads, and deeper and more varied learning; a monthly which shall, by the power of language, make fact more interesting than fiction; whose every page shall announce some physical, or moral, or social, or natural truth, in a manner so pleasing, or so profound, yet clear, as shall make it looked for, from month to month, with the eagerness with which, now, the drunken revellers in fiction anticipate the coming of the next installment of crazing and corrupting stimulants; for that the vast mass of fictitious reading found in the newspapers and magazines of the day, does but craze and corrupt the heads and hearts of those who indulge in them, can not be denied. If this suggestion shall prove to be a first foundation stone, the nucleus around which shall gather men of a true religion, as well as men of might, who shall build upon it, it is well.

